# Nine years of pediatric gunshot wounds: A descriptive analysis

**DOI:** 10.1016/j.pmedr.2022.101890

**Published:** 2022-07-05

**Authors:** Grant Woodruff, Lilly Palmer, Emily Fontane, Colleen Kalynych, Phyllis Hendry, Arielle C. Thomas, Marie Crandall

**Affiliations:** a University of Florida College of Medicine Jacksonville, Department of Surgery, USA; b East Carolina University Department of Surgery, Greenville, NC, USA; c Mary Bridges Children’s Hospital, Pediatric Emergency Medicine, Tacoma, WA, USA; d University of Florida College of Medicine Jacksonville, Department of Emergency Medicine, Jacksonville, FL, USA; e University of Florida College of Medicine Jacksonville, Educational and Development Research, Jacksonville, FL, USA; f American College of Surgeons, Chicago, IL, USA

**Keywords:** Pediatric trauma, Epidemiology, Firearm injury, Violence prevention

## Abstract

Pediatric firearm violence carries significant morbidity and mortality. Studies targeting children ≤14 years are limited. Our goal was to study the distribution and determinants of GSWs in the pediatric population. We performed a retrospective review of children ≤14 years presenting with GSWs at this level 1 trauma center. This cohort was split into younger children, 0–12 years, and older children, 13–14 years. Summary and bivariate statistics were calculated using Stata v10. 142 patients (68.3% black, 76.7% male) were identified. Injuries more often occurred at home (39.6%) by family or friends (60.7%). Older children often suffered handgun injuries (85.5%) and more often were sent immediately to the OR on presentation (29.2%). Younger children more often suffered from air-gun (50%) and pistols (40%). Younger children more commonly had blood transfusions (9.4%) compared to exploratory laparotomy in older children (13.5%). The most common disposition from the ED was home (36.2%). Descriptive data entailing incident specifics such as time of injury and CPS involvement were frequently missing in the healthcare record. Older children were more likely to be injured by strangers, have longer lengths of stay especially associated with surgical operations, and have a disposition of immediate arrest compared to their younger cohort. Consequently, this group may benefit from interventions typically aimed at older patients such as violence intervention programs. When available, differences in demographics and outcomes were identified which could shape novel prevention strategies for firearm injury.

## Highlights

• Firearm injury increases with age with previous studies examining ages 0–14 vs. 15–18.
• There is poor documentation in the literature of key demographic details surrounding the injury.
• There are important demographic differences that exist between older adolescent (13–14 years) and younger children (0–12 years).
• Adolescents aged 13 years and older have similar profile to older adolescents/teens and may benefit from interventions such as violence intervention programs.

## 1 Introduction

The United States (US), as compared to other high-income countries, has the highest rates of gunshot-related deaths in all age groups (Richardson and Hemenway, 2011, Grinshteyn and Hemenway, 2019). Firearm violence amongst the pediatric population in the US is a significant cause of morbidity and mortality. Despite an overall decline in incidence of pediatric firearm violence events, it remains an important public health concern with over 13,000 pediatric firearm events treated in US emergency departments (EDs) annually (Jones et al., 2019).

Gunshot wounds (GSWs) are associated with one of the highest fatality rates (20%) of all injury mechanisms and even higher (26%) in the youngest pediatric population (0–10 years) (Nance et al., 2013). In 2019, the Center for Disease Control and Prevention (CDC) ranked homicide by firearm injury as the 4th leading cause of death in US children 1–14 years of age with a reported 259 firearm related deaths (2021). According to the National Trauma Data Bank 2016 Pediatric Report, Gunshot Injury (GSI) incidence was highest in children 13 and 14 years of age with case fatality rates of 11.2% across all pediatric age groups (2016).

In 2013, the Institute of Medicine (IOM) recognized a need to develop firearm violence prevention programs (Leshner et al., 2013). However, there remains a significant deficit regarding availability of data that could subsidize an evidence-based approach to firearm violence prevention (Hemenway and Miller, 2013). While population-based epidemiological studies regarding GSWs in youth do exist, they are limited with respect to the youngest pediatric population (0–14 years of age). It has been postulated that GSW risk factors and demographics differ between young children and adolescents (15–18 years old) (Cunningham et al., 2009, Andrews et al., 2022, Cheng and Burjonrappa, 2022). Further study is needed to adequately describe intent, identify epidemiological trends, and improve injury surveillance in younger vs. older adolescent patients. Many existing studies cite the use of local databases or vital statistics to establish reported epidemiological trends. (Urrechaga et al., 2021) Unfortunately, these lack the data necessary to develop novel firearm violence prevention strategy and/or contribute to prospective study design. In contrast, urban EDs are an underutilized but potentially fruitful data source for GSW demographics in this patient population and can provide details such as timing of injury around school, injury intent, and role of individual in the injury (Davis et al., 2013, Senger et al., 2011, Evans et al., 2020, Tatebe et al., 2021).

The objective of this study is to build upon previous work done by our group and retrospectively review the data available through an urban ED to describe the distribution and determinants of GSWs, as well as injury and treatment patterns common to this youngest group of patients. The data used were part of a continuing longitudinal data set which included earlier data that previously underwent a similar analysis (Hendry et al., 2014). The conclusions drawn from this dataset can help stimulate the development of future firearm violence prevention methods targeted by age range subgroups in the pediatric trauma population.

## 2 Methods

Following Institutional Review Board approval, patients ages 0–14 years who sustained one or more GSWs and presented to this Level 1 trauma center between January 2005 and December 2013, were identified using the trauma registry, hospital logs, and corresponding ICD-9 billing codes.

A retrospective review of the electronic health record, including Emergency Medical Service (EMS) records, ED, and inpatient documentation, was completed. No patients with the aforementioned characteristics were excluded from review unless electronic medical records could not be found. Data that were collected included patient demographics, social characteristics, injury pattern, treatment course, and ED disposition. Additional descriptive details surrounding the firearm violence event (weapon, shooter, shooter’s relationship to patient, and intent) were noted when available.

All data were recorded using Research Electronic Data Capture (REDCap). Summary statistics were calculated for categorical variables. Since 13–14 years of age historically has carried the highest GSW incidence in the pediatric population, the cohort was split into two age groups for analysis: younger children, 0–12 years, and older children, 13–14 years of age. A Chi-Square Test of Independence was conducted to examine whether gender, race, type of gun, intent, and disposition were independent of age. Two-sample t-tests with equal variance were performed to compare mean hospital length of stay (LOS) and shooter age for both age groups. Due to the lower-than-expected availability of GSW specific data, percentages presented in the results are according to the available data, not the entire cohort.

## 3 Results

A cohort of 142 children were identified for inclusion (Table 1). There were 53 patients aged 0–12 years and 89 patients aged 13–14 years. The majority of GSW victims were found to be male (76.7%) and African American (68.3%). When available, the average median household income for incident location was $39,350 (n = 32). A majority (89.4%, n = 127) were noted to have insurance (Medicaid, Tricare or Commercial). The entire younger population was found to have insurance; however, in the older age group, 15 patients were reported as self-pay (16.9%, n = 89). When documented, none of the children in the younger cohort had a prior history of abuse, but in the older population, 26.9% of patients had a previously reported history of abuse.

**Table 1:** Comparison of pediatric age groups (0–12 years) vs. (13–14 years).

**Characteristics**		**Ages 0**–**12**	**Ages 13**–**14**	***p-*value**
		**(n=53)**	**(n=89)**	
**Race**	African American	30 (56.6%)	67 (75.3%)	0.14
	Caucasian	20 (37.7%)	17 (19.1%)	
	Native American/Pacific Islander	0 (0%)	1 (1.1%)	
	Other	2 (3.8%)	3 (3.4%)	
	Not Documented	1 (1.9%)	1 (1.1%)	
**Sex**	Male	38 (71.7%)	71 (79.8%)	0.27
	Female	15 (28.3%)	18 (20.2%)	
**Location of**	Home	22 (41.5%)	18 (20.7%)	0.017
**Assault**	Street	7 (13.2%)	33 (37.9%)	
	Recreational Area	0 (0%)	3 (3.4%)	
	Hunting	1 (1.9%)	2 (2.3%)	
	Drive By	2 (3.8%)	2 (2.3%)	
	Other	6 (11.3%)	5 (5.7%)	
	Not Documented	15 (28.3%)	24 (27.6%)	
**Shooter**	Stranger	2 (3.8%)	18 (20.2%)	0.013
	Mother	1 (1.9%)	0 (0%)	
	Father	2 (3.8%)	0 (0%)	
	Sibling	4 (7.5%)	2 (2.2%)	
	Other Family Member	0 (0%)	3 (3.4%)	
	Friend	4 (7.5%)	6 (6.7%)	
	Other	7 (13.2%)	5 (5.6%)	
	Self	1 (1.9%)	1 (1.1%)	
	Not Documented	32 (60.4%)	54 (60.7%)	
**Type of Weapon**	Pistol	12 (22.6%)	53 (59.6%)	<0.001
	Shotgun	2 (3.8%)	2 (2.2%)	
	Air-gun	15 (28.3%)	7 (7.9%)	
	Other	1 (1.9%)	0 (0%)	
	Not Documented	23 (43.4%)	27 (30.3%)	
**Site of Injury**	Head	11 (20.8%)	14 (15.7%)	0.012
	Neck	4 (7.5%)	6 (6.7%)	
	Trunk	34 (64.2%)	37 (41.6%)	
	Extremities	13 (24.5%)	38 (42.7%)	
	Multiple	15 (28.3%)	7 (7.9%)	
**Trauma Disposition**	Mortality	0 (0%)	5 (5.6%)	0.015
	OR	8 (15.4%)	26 (29.2%)	
	ICU	16 (30.8%)	12 (13.5%)	
	Floor	8 (15.4%)	10 (11.2%)	
	Jail	0 (0%)	2 (2.2%)	
	Home	17 (32.7%)	34 (38.2%)	
	Transfer	3 (5.8%)	0 (0%)	
**History of Abuse**	Yes	0 (0%)	7 (8.2%)	0.036
	No	7 (13.7%)	19 (22.4%)	
	Not Documented	44 (86.3%)	59 (69.4%)	
**Diagnosis of ADHD**	Yes	5 (9.4%)	3 (3.4%)	0.25
	No	48 (90.6%)	86 (96.6%)	
**Child Protective Services notification**	Yes	9 (16.9%)	6 (6.7%)	<0.01
	No	3 (5.7%)	53 (58.9%)	
	Undocumented	41 (77.4%)	30 (33.3%)	

Approximately half of the patients presented during the day shift (0600–1800 h). Most patients were transported by EMS (84.4%, n = 114). Disposition from the ED or trauma center was available for 141 patients: this included discharge to home (36.2%), transfer to the operating room (24.1%), Intensive Care Unit (ICU) admission (19.9%), non-ICU admission (14.9%), discharge into law enforcement custody (1.4%), and transfer to the morgue (0.7%). Disposition from the trauma center significantly varied by age group (p = 0.015). In both groups the most common disposition was to home. This was followed by admission to the ICU (30.8%, n = 52) for the younger cohort, and immediate transfer to the operating room (29.2%, n = 89) for the older group. The older group had several patients dispositioned to the morgue or transferred directly into the custody of law enforcement, whereas the younger group had no deaths in the emergency department or immediate arrests.

The primary injury sites (Table 2), in order of decreasing frequency, were trunk (38.7%), extremities (35.9%), head (17.6%), and neck (7.04%) with 22 patients (15.5%) sustaining GSWs at multiple sites. Common procedures included intubation (18.3%), blood transfusions (7%) and exploratory laparotomy (11.3%). The overall mortality rate was 3.5%, including death during ED and/or hospital admission. Fatal GSWs occurred in only 5 patients. Of the five patients who died, two patients died of torso trauma. Neither had vital signs upon arrival. The other three patients died of severe traumatic brain injuries secondary to GSWs to the head. Mean hospital LOS was significantly longer (8.15 ± 1.45 days) for older children, approximately double the LOS (4.16 ± 0.76 days) of the younger patients (p = 0.04). The most common procedure performed in both cohorts was wound irrigation and dressing (35.9% in the younger population, 41.6% in the older population). When comparing the age groups, other common procedures performed in the younger population included intubation (17%) and blood transfusion (9.4%), while the older population included intubation (19.1%) and exploratory laparotomy (13.5%). Both cohorts also had a variety of procedures performed defined under “other” procedures (34% in the younger vs 19.1% in the older). These procedures were widely varied and included debridement and irrigations, orthopedic interventions, and neurosurgical interventions. When comparing the age groups, they underwent a similar variety of procedures with notable differences including: 2 patients in the younger population requiring neurosurgical intervention (1 craniotomy, 1 craniectomy) vs 0 patients in the older population requiring neurosurgical intervention, 5 patients in the younger population undergoing foreign body removal vs 1 patient in the older population, and 3 patients requiring operative orthopedic fixation in the older population vs 0 patients in the younger.

**Table 2:** Procedures undergone by pediatric GSW victims.

**Procedures**	**Ages 0**–**12 (n=53)**	**Ages 13**–**14 (n=89)**
Intubation	9 (17%)	17 (19.1%)
Chest tube	1 (1.9%)	1 (9%)
Central line	0 (0%)	1 (4.5%)
Blood transfusion	5 (9.4%)	5 (5.6%)
Sedation	4 (7.6%)	1 (1.1%)
Wound irrigation/Dressing	19 (35.9%)	37 (41.6%)
Exploratory laparotomy	4 (7.6%)	12 (13.5%)
Wound care	4 (7.6%)	5 (5.6%)
Splint	1 (1.9%)	4 (4.5%)
Other	18 (34%)	17 (19.1%)

Shooter information and intent was rarely provided in the chart. When documented, the majority of GSWs were not intentional (78.7%, n = 47). The identity of the shooter was recorded in 56 of the 142 cases of which 60.7% of shooters were found to be family members or friends; 35.7% of shooters were unknown to the patient. Comparing the relationship of the shooter to the victim, younger children were more likely to be injured by a known family member or friend (85.7%, n = 21), while older children were more likely to be injured by strangers (51.4%, n = 35; p = 0.003). When the location of the shooting was known (n = 101), the patient’s home was most often the site of the shooting (39.6%). However, when available (n = 71), documentation showed that child protective services were notified in only 21.1% of cases and were significantly more likely to be notified of the child’s involvement in a firearm violence event if the child was <12 years of age (p < 0.001).

The specific weapon involved was not documented in 50 cases (35%). For the cases when data were available, 65 firearm violence events involved handguns (45.8%), 4 involved shotguns (2.8%), and 22 involved air-guns (15.5%). Older children were more likely to be injured by handguns versus other weapons (85.5%, n = 62, OR 1.5, p < 0.001), while the younger population was involved with air-gun injury (50%, n = 30) and pistols (40%, n = 30). The most frequently reported caliber of bullet used was a 0.9 mm (20.7%, n = 29) followed by 0.22 mm (13.8%, n = 29).

## 4 Discussion

The demographic data of our youngest pediatric patients were generally consistent with the studied adolescent patient population: patients were predominantly male and black (Senger et al., 2011, Hendry et al., 2014, Tseng et al., 2018, Fowler et al., 2017). Injury sites were diverse and varied in severity as evidenced by our intubation and transfusion rates. This study further highlights the differences in injuries between older and younger children. Older children were more likely to be injured by strangers, have longer lengths of stay especially associated with surgical operations, be injured by handguns, and have a disposition of immediate arrest compared to their younger cohort. It is important that physicians recognize the differences between older and younger patients’ etiology of GSI as they potentially have different injury prevention requirements.

The differences between older adolescent and younger patients indicate that older patients are potentially a different population and should be treated as thus. The risk factors for injury starting at age 13 could potentially diverge from the factors associated with injury between ages 0–12 and are more related to injury noted in the adult population. This is also consistent with data from the National Trauma Database Pediatric report that shows a doubling of firearm injury incidence between ages 12 and 13 (82 vs. 153) which continues to increase with each subsequent year (National Trauma Data Bank, 2016). Violence intervention programs, historically focused on adult firearm trauma, are structured to target the risk factors underlying firearm injury which are varied but include limited education, lack of economic opportunities, and residing in an area of socioeconomic disadvantage (Dicker et al., 2017). These programs, typically hospital or community based, allow for a connection of these at-risk individuals to case managers that create individualized plans and follow them in the outpatient setting. This could intervene in the cycle of violence and recidivism that often plagues individuals who are victims of firearm trauma.

Child protective services were more likely to be notified in the younger pediatric population, although when known, child protective service staff was only notified in 21.1% of our cases. We believe this number to be lower than reported. Child abuse was also overwhelmingly undocumented. There was no history of abuse documented in the younger cohort, while the older cohort had 7 children with a documented history of prior abuse and 59 children with an undocumented history. In all six cases where CPS was notified for older adolescents, the shooter was known to the victim and/or the victim’s family. Furthermore, when the shooter was known to be a stranger (n = 20) and when CPS notification status was recorded for those patients (n = 19), none of those incidents were reported to CPS (0%). In the future, prior history of child abuse should be further investigated to determine if these at-risk adolescents have higher incidence of GSWs compared to those with no history of child abuse. Child protective services should also be notified in all cases of firearm injury and not only when the shooter is known to the family. This highlights a major limitation to our study and the need for proper documentation within the health record. At the very least, the Department of Child Services should be notified and documented consistently in the medical record.

Another limitation to our study is that the date of injury was missing from our data set. As a result, seasonal variations in gun violence were not available for comparison. Based on previous studies, firearm violence is most likely to occur during the summer months and at night (Veenstra et al., 2015). However, Tatebe et al. noted that 43.6% of firearm injuries happened during times of school or curfew and 22% occurred during the after-school period. Our results demonstrated approximately half of our population presented during the day shift and half during night shift, although exact time of day was not available for review. These limitations represent potential targets for future studies directed toward implementing effective prevention programs. Future studies are needed to create prevention strategies to target higher incidence seasons and times of day.

Our study population had an unusually low mortality rate compared to historic data. Previous studies report fatality rates ranging from 9 to 20% in the pediatric population (ages 0–18) (Andrews et al., 2022, Fowler et al., 2017, Hatchimonji et al., 2020, Theodorou et al., 2022). The mortality rate published by the National Trauma Database in the 2016 Pediatric Report was 11.2% (National Trauma Data Bank, 2016). We postulate that our lower mortality rate is due to the exclusion of patients found dead at the scene from our Trauma Regional Advisory Council (RAC). Another cause could be survivorship bias, as 19 patients (13.5%) were transferred to our center from an outside hospital.

Incident related data, including weapon choice and relationship of shooter to patient, was too infrequently documented to truly define an epidemiological trend; however, the few cases where this was documented do reveal several pertinent findings. The majority of firearm violence incidents occurred in areas of lower income neighborhoods and at home regardless of intent, which is consistent with the current literature (Agran et al., 2001, Miller et al., 2002, Lee et al., 2013). The majority of cases where shooter demographics were available, noted the shooter to be family or known to the patient, especially in the younger population. The availability of firearms and stringency of legislation has been linked to increased unintentional firearm deaths, homicides, and suicides (Tseng et al., 2018, Madhavan et al., 2019). It has also been shown that in households with firearms, the use of gun locks, safe storage devices, and safe ammunition storage techniques have been associated with decreases in firearm injury (Violano et al., 2018, Grossman et al., 2005). Utilizing data, like what was found in this study, may help in identifying those individuals at the highest risk for pediatric gun violence. Once identified, education and distribution of affordable gun locks, storage devices and teachings for safe ammunition storage techniques may prove beneficial in reducing future firearm injuries.

Conclusions drawn from this study are limited as it is retrospectively collected data, and more importantly incomplete data. This study was limited to patients who survived to receive hospital services. Furthermore, the sparse and infrequent documentation of incident related information (i.e. type of weapon, relationship of shooter to patient, motivation/intent, etc), illustrates the dire need for a change in the medical record documentation methods as it relates to GSWs and more specifically pediatric GSWs. While these incident details would be available within law enforcement records, we as physicians do not have access. Ideally, EMS and law enforcement documentation surrounding a GSW should be made available to physicians as a part of the patient’s electronic health record. Until that is possible, a more comprehensive and synergistic approach to documentation of incident data needs to occur, so that further study can establish true epidemiological trends. Conclusions can then stimulate novel firearm violence prevention strategies specifically targeted to this young pediatric population.

## 5 Declarations

• Funding (information that explains whether and by whom the research was supported): none
• Ethics approval (include appropriate approvals or waivers): University of Florida Institutional Review Board
• Consent to participate (include appropriate consent statements): N/A
• Consent for publication (consent statement regarding publishing an individual’s data or image): N/A
• Availability of data and material (data transparency): Available upon request
• Code availability (software application or custom code): Available upon request

## Declaration of Competing Interest

The authors declare that they have no known competing financial interests or personal relationships that could have appeared to influence the work reported in this paper.
